# ‘Only Fathers Smoking’ Contributes the Most to Socioeconomic Inequalities: Changes in Socioeconomic Inequalities in Infants’ Exposure to Second Hand Smoke over Time in Japan

**DOI:** 10.1371/journal.pone.0139512

**Published:** 2015-10-02

**Authors:** Junko Saito, Takahiro Tabuchi, Akira Shibanuma, Junko Yasuoka, Masakazu Nakamura, Masamine Jimba

**Affiliations:** 1 Department of Community and Global Health, Graduate School of Medicine, The University of Tokyo, 7-3-1 Hongo, Bunkyo-ku, Tokyo, 113–0033, Japan; 2 Center for Cancer Control and Statistics, Osaka Medical Center for Cancer and Cardiovascular Diseases, 3–3 Nakamichi 1-chome, Higashinari-ku, Osaka, 537–8511, Japan; 3 Health Promotion Research Center, Institute of Community Medicine, Japan Association for Development of Community Medicine, Todofuken Kaikan Bldg, 15th Floor, 2-6-3 Hirakawa-cho, Chiyoda-ku, Tokyo, 102–0093, Japan; TNO, NETHERLANDS

## Abstract

**Background:**

Exposure to second hand smoke (SHS) is one of the major causes of premature death and disease among children. While socioeconomic inequalities exist for adult smoking, such evidence is limited for SHS exposure in children. Thus, this study examined changes over time in socioeconomic inequalities in infants’ SHS exposure in Japan.

**Methods:**

This is a repeated cross-sectional study of 41,833 infants born in 2001 and 32,120 infants born in 2010 in Japan from nationally representative surveys using questionnaires. The prevalence of infants’ SHS exposure was determined and related to household income and parental education level. The magnitudes of income and educational inequalities in infants’ SHS exposure were estimated in 2001 and 2010 using both absolute and relative inequality indices.

**Results:**

The prevalence of SHS exposure in infants declined from 2001 to 2010. The relative index of inequality increased from 0.85 (95% confidence interval [CI], 0.80 to 0.89) to 1.47 (95% CI, 1.37 to 1.56) based on income and from 1.22 (95% CI, 1.17 to 1.26) to 2.09 (95% CI, 2.00 to 2.17) based on education. In contrast, the slope index of inequality decreased from 30.9 (95% CI, 29.3 to 32.6) to 20.1 (95% CI, 18.7 to 21.5) based on income and from 44.6 (95% CI, 43.1 to 46.2) to 28.7 (95% CI, 27.3 to 30.0) based on education. Having only a father who smoked indoors was a major contributor to absolute income inequality in infants’ SHS exposure in 2010, which increased in importance from 45.1% in 2001 to 67.0% in 2010.

**Conclusions:**

The socioeconomic inequalities in infants’ second hand smoke exposure increased in relative terms but decreased in absolute terms from 2001 to 2010. Further efforts are needed to encourage parents to quit smoking and protect infants from second hand smoke exposure, especially in low socioeconomic households that include non-smoking mothers.

## Introduction

Exposure to second hand smoke (SHS) is one of the major causes of premature death and disease among children[1]. The majority of SHS exposure in children occurs in homes or cars because their parents smoke, and 40% of children worldwide are regularly exposed to SHS indoors[2]. Although an ideal solution to protect children from exposure to SHS is parents’ cessation of smoking, this is often not achievable[3,4]. ‘Home smoking bans’ might be an alternative and realistic strategy to reduce SHS exposure among children[5], although this is not a perfect solution[6].

The level of SHS exposure in children differs based on the parents’ socioeconomic status (SES), with children in lower SES groups more likely to be exposed to SHS in their homes or cars than children in higher SES households[7,8]. Although social inequalities in the prevalence of adult smoking are widening in some European countries[9], there are limited studies focusing on the changes in SHS exposure inequalities in children over time. The available studies have shown mixed results. While the overall prevalence of SHS exposure in children decreased, socioeconomic inequalities in children’s SHS exposure increased in the USA[10], and remained the same in Australia and Denmark[11,12]. However, children younger than two years old have rarely been studied independent of their older children despite they are more susceptible to the risks associated with SHS exposure[13]. Moreover, although mothers are more likely to be motivated to protect children from SHS exposure than fathers[3], it is not known how the combination of parental indoor smoking (i.e., only father smokes indoors, only mother smokes indoors, and both parents smoke indoors) contributes to these inequalities over time. Examining which combinations of parental indoor smoking drive inequalities the most would help to prioritize tobacco control policies or interventions to reduce children’s SHS exposure.

Japan has significantly lagged behind in legislative tobacco control measures, despite having signed the World Health Organization’s (WHO) Framework Convention on Tobacco Control in 2004[14,15]. No national law prohibits smoking in public places in Japan. Although two prefectures adopted an ordinance to restrict smoking in indoor public places in 2009 and 2010[16,17], it is not mandated that all public places provide smoke-free environments[17]. Additionally, the tax rate of cigarettes in Japan rose from 61% to 65% of the retail price during 1998 to 2010[18]; however, it has not yet reached the criteria set by the WHO (at least 70%)[19].

Many children are still exposed to SHS in Japan. While the prevalence of adult smoking decreased from 45.9% in men and 9.9% in women in 2001 to 32.2% in men and 8.4% in women in 2010[20,21], approximately 40% of infants live with a smoking father and 14% live with a father who smokes indoors[22]. Moreover, socioeconomic inequality in SHS exposure among children is inferred in Japan, although the evidence is limited. A study using nationally representative data in Japan demonstrated a significant relationship between parental smoking and household income[23]. Children in the lowest income households are more likely to suffer from asthma compared with those in the highest income households[24], and parental indoor smoking increases and exacerbates children’s asthma[25]. Given these findings, we hypothesise that SHS exposure is higher among children in low-SES households in Japan. However, the change over time regarding the effect of SES on SHS exposure in children has not been studied in Japan.

Thus, we examined the magnitude of inequalities and changes in the magnitude of inequalities in SHS exposure in infants from 2001 to 2010. We hypothesized that the inequalities in SHS exposure based on parental income and education in Japan have remained the same during a recent 10-year period.

## Materials and Methods

### Study sample

We used data from the Longitudinal Survey of Newborns in the 21^st^ Century, which was a national survey conducted by the Ministry of Health, Labour, and Welfare, Japan. This large panel study has two cohorts (infants who were born in 2001 or 2010). Data were used with permission from the Ministry of Health, Labour, and Welfare, Japan. Baseline data of both cohorts were used in this study, for all infants born in Japan during January 10–17, 2001 or July 10–17, 2001 for the first cohort (n = 53,575) and May 10–24, 2010 for the second cohort (n = 43,767), based on the birth registry. The respondents who returned the questionnaire to the Ministry were considered to have agreed to participate in the study. A detailed description of the cohort survey is available elsewhere[26].

The response rate for the first self-administered questionnaire, which was mailed to all households when the infants were 6 months old, was 87.8% (n = 47,015/53,575) for the first cohort and 88.1% (n = 38,554/43,767) for the second cohort. The response rate for the second questionnaire, which was mailed to participants of the first survey when their children reached 18 months old, was 82.0% (n = 43,925/53,575) for the first cohort and 76.2% (n = 33,356/43,767) for the second cohort. We restricted study participants to infants whose parents lived together at baseline, which led to exclusion of 923 for the first cohort and 686 for the second cohort. In addition, infants lacking parental age (151 for the first cohort, 103 for the second cohort) and parental smoking status (1,021 for the first cohort, 454 for the second cohort) were excluded. In the final analyses, 41,833 (78.1% of the initial cohort) and 32,120 (73.4% of the initial cohort) respondents for the first and second cohorts, respectively, were included. This study was approved by the Research Ethics Committee of the Graduate School of Medicine at The University of Tokyo, Japan.

### Second hand smoke exposure in infants

Parental indoor smoking behaviour was used as a measurement of SHS exposure in infants[11]. Although this is a proxy measurement, parental indoor smoking is significantly associated with biochemically measured SHS exposure among children[27]. The parents in the baseline survey were asked whether the father and/or mother smoked, and, if yes, they were asked whether they smoked indoors. Then, we combined the responses for the smoking behaviour of both parents to create parental smoking (at least one parent smoked vs. neither parent smoked) and parental indoor smoking (at least one parent smoked indoors vs. neither parent smoked indoors) variables.

### Socioeconomic indicators

We used income and education as SES indicators. For income, equivalent household income was calculated by adjusting for the square root of the number of persons living in the household and categorized into quartiles for each survey. Because the education question was only included in the second survey (2002 for the first cohort and 2011 for the second cohort), those data were used for education level (highest completed level) and categorized into four groups: less than high school graduate, high school graduate, some college, or university graduate or higher. Then, we combined the parental education level of the mother and father as follows: both are high school graduates or less, one is a college graduate and the other is a college graduate or less, only one is a university graduate, or both are university graduates or higher.

### Statistical analyses

We calculated the prevalence of SHS exposure in infants based on SES by survey year. The prevalence in 2010 was adjusted by the average parental age in 5-year age groups using a direct method and the parental age distribution in 2001 as the base. Then, we used two methods to examine the change in inequality from 2001 to 2010. First, we compared the inequality indices between the two periods, using both absolute and relative indices, which is strongly recommended in health equality research to avoid biased judgments by readers[28]. For absolute measures, the rate difference and slope index of inequality (SII) with 95% confidence intervals (CIs) were estimated. The rate difference measures the absolute difference in indoor smoking prevalence between the lowest and highest SES groups. For relative measures, the odds ratio (OR) and relative index of inequality (RII) with 95% CIs were estimated. The OR is the ratio of the odds of indoor smoking in the lowest compared with the highest SES group and was estimated using logistic regression models, controlled for infant’s sex, father’s and mother’s ages, and the SES variables (either income or education). To avoid overadjustment (i.e., control of an intermediate variable on a causal path from exposure to outcome[29]), we did not adjust for variables that would possibly mediate the relationship between SES and SHS exposure in infants (e.g., the number of cigarettes parents smoke per day and the spouse’s smoking status). We chose SII and RII as inequality indices because the sample sizes of the four parental education groups were quite different across groups in both years. The SII and RII were estimated as regression-based measures of SHS that took into account the distributions of the sample in each SES group and the entire distribution of the SES groups over time[30]. The SII can be interpreted as the estimated absolute difference in the prevalence of SHS between infants with the highest and lowest SES. The RII is derived by dividing the SII by the mean prevalence of SHS exposure and can be interpreted as the estimated proportionate difference, rather than the absolute difference[30,31].

Second, we determined the change in the prevalence over time for each SES group separately using the pooled data in 2001 and 2010. For each SES group, the rate difference and percentage change were calculated. Further, the coefficient of interaction terms between SES (income or education) and year of survey using logit regression models were estimated, controlled for infant’s sex, father’s and mother’s ages, and SES variables. Then, we compared the coefficient of interaction terms (with 95% CIs) across SES groups to examine whether changes in inequality were different by SES.

Further, we calculated the prevalence and the magnitude of inequalities (SII and RII) based on income level by parental indoor smoking behaviour (only father smokes indoors, only mother smokes indoors, and both parents smoke indoors). Then, we calculated the proportion to the total SII represented by each parental indoor smoking behaviour to examine the contribution for the total income inequality[32]. We did not calculate an educational SII by parental indoor smoking behaviour because the categorization of education level between father/mother indoor smoking and parental indoor smoking was not the same.

As a sub-analysis, we examined the changes in inequalities in parental smoking over time to examine whether they were comparable to the changes in SHS exposure in infants. We also examined the changes in inequalities in parental smoking over time for each parental smoking behaviour and compared the changes with those in SHS exposure in infants. Most of the analyses were conducted using STATA 13 (StataCorp LP; College Station, TX, US); the SII and RII calculations were conducted using HD*calc (version 1.2.4; National Cancer Institute, US)[33].

## Results

Table 1 shows the characteristics of the study population by survey year and distribution of infants living with smoking parents and exposed to SHS in 2001 and 2010. The average parental age was 31.1 years old (standard deviation [SD], 4.55; range, 17.5–54.0) in 2001 and 32.6 years old (SD, 4.69; range, 20.0–59.5) in 2010 (data not shown). The percentage of infants exposed to SHS declined from 36.8% in 2001 to 14.4% in 2010.

**Table 1:** Characteristics of the study population from the Longitudinal Survey of Newborns in the 21^st^ Century in Japan, by survey year.

	2001		2010	
	n = 41,833	%	n = 32,120	%
**Equivalent household income**				
Quartile 1 (highest)	9,827	23.5	7,565	23.6
Quartile 2	10,236	24.5	7,541	23.5
Quartile 3	9,503	22.7	7,837	24.4
Quartile 4	9,713	23.2	7,131	22.2
Missing	2,554	6.1	2,046	6.4
**Parental education level**				
Both are university graduates or higher	4,572	11.0	6,460	20.1
Only one is a university graduate	11,832	28.3	9,720	30.3
One is a college graduate and the other is a college graduate or less	12,555	30.0	9,570	29.8
Both are high school graduates or less	12,473	29.8	6,111	19.0
Missing	401	1.0	259	0.8
**Infant sex**				
Boy	21,754	52.0	16,548	51.5
Girl	20,079	48.0	15,572	48.5
**Father's age (years)**				
≤24	2,715	6.5	1,284	4.0
25–29	11,159	26.7	6,310	19.7
30–34	15,413	36.8	11,204	34.9
35–39	8,675	20.7	9,101	28.3
≥40	3,871	9.3	4,221	13.1
**Mother's age (years)**				
≤24	4,242	10.1	2,053	6.4
25–29	15,210	36.4	8,304	25.9
30–34	16,170	38.7	12,489	38.9
35–39	5,459	13.1	7,803	24.3
≥40	752	1.8	1,471	4.6
**Living with smoking parent(s)** ^**a**^				
Yes	26,453	63.2	13,406	41.7
No	15,380	36.8	18,714	58.3
**Exposed to second hand smoke** ^**a**^ ^**,**^ ^**b**^				
Yes	15,403	36.8	4,619	14.4
No	26,430	63.2	27,501	85.6

^a^ The number and prevalence in 2010 were weighted for the average parental age in 5-year age groups using a direct method and the age distribution in 2001 as the base.

^b^ Exposure to second hand smoke was measured by self-reported parental indoor smoking behaviour.

Table 2 shows the prevalence of SHS exposure and the magnitude of income and educational inequalities in SHS exposure in infants in 2001 and 2010. The prevalence of SHS exposure in infants in the lowest and highest income groups was 47.9% and 24.7% in 2001 and 22.6% and 7.0% in 2010, respectively. Income and educational inequalities in SHS exposure in infants existed; for example, in 2010, the rate difference and the SII in the prevalence of infants’ SHS exposure based on income were 15.6 and 20.1, demonstrating greater prevalence in the lowest income group compared with the highest group; the OR indicated a 1.89 times higher odds of infants’ SHS exposure in the lowest income group than in the highest income group; and the RII indicated that a move from the highest to the lowest income group was associated with a 147% increase in the prevalence of SHS exposure.

**Table 2:** Prevalence of second hand smoke (SHS) exposure in infants and magnitude of inequalities in SHS exposure in infants according to income and educational level by survey year.

	Prevalence of SHS exposure in infants (%)				
	2001	2010^b^	Rate difference (%point) (2010–2001)	% change ([2010–2001]/2001)	Coefficient (95% CI)^c^ (Income × year)
**Equivalent household income**					
Quartile 1 (highest) (ref)	24.7	7.0	-17.7	-71.7	-1.32 (-1.42 to -1.22)
Quartile 2	33.1	10.7	-22.4	-67.7	-1.30 (-1.39 to -1.21)
Quartile 3	41.0	14.9	-26.1	-63.7	-1.29 (-1.37 to -1.21)
Quartile 4 (lowest)	47.9	22.6	-25.3	-52.8	-1.11 (-1.18 to -1.04)
Rate difference (lowest—highest) (% point)	23.2	15.6			
SII (95% CI)	30.9 (29.3 to 32.6)	20.1 (18.7 to 21.5)			
Odds ratio^a^ (95% CI)	1.69 (1.58 to 1.81)	1.89 (1.68 to 2.12)			
RII (95% CI)	0.85 (0.80 to 0.89)	1.47 (1.37 to 1.56)			
**Parental education level**					
Both are university graduates or higher (highest) (ref)	14.8	4.0	-10.8	-73.0	-1.46 (-1.61 to -1.31)
Only one is a university graduate	26.2	8.9	-17.3	-66.0	-1.36 (-1.44 to -1.27)
One is a college graduate and the other is a college graduate or less	40.0	16.0	-24.0	-60.0	-1.27 (-1.34 to -1.20)
Both are high school graduates or less (lowest)	51.5	28.1	-23.4	-45.4	-1.06 (-1.13 to -0.99)
Rate difference (lowest—highest) (% point)	36.7	24.1			
SII (95% CI)	44.6 (43.1 to 46.2)	28.7 (27.3 to 30.0)			
Odds ratio^a^ (95% CI)	4.65 (4.23 to 5.10)	6.58 (5.67 to 7.64)			
RII (95% CI)	1.22 (1.17 to 1.26)	2.09 (2.00 to 2.17)			

CI, confidence interval; SII, slope index of inequality; RII, relative index of inequality

^a^ Adjusted by father’s age, mother’s age, infant sex, and socioeconomic status indicators (either income or education)

^b^ The prevalence in 2010 was weighted for the average parental age in 5-year age groups using a direct method and the age distribution in 2001 as the base.

^c^ Adjusted by father’s age, mother’s age, infant sex, and socioeconomic status indicators (both income and education)

Regarding changes over time, the prevalence of SHS exposure decreased in all SES groups from 2001 to 2010. The absolute measures of inequality (rate difference and SII) indicated that the magnitude of income and educational inequalities in SHS exposure among infants decreased from 2001 to 2010, while the relative measures of inequality (OR and RII) increased. For instance, from 2001 to 2010, the SII for income decreased from 30.9 to 20.1, while the RII for income increased from 0.85 to 1.47. In the comparison of the SES groups, the lowest SES group showed the largest absolute decrease (-25.3 percentage points) in prevalence of SHS exposure, while the relative decrease was the smallest (-52.8 percentage change), supporting the results that income and educational inequalities increased in relative terms but decreased in absolute terms over time. The interaction analysis resulted in statistically significant coefficient of interaction terms in each SES group and negatively larger terms with increasing SES group (both income and parental education). The sub-analysis of inequality changes showed a much smaller relative decrease (percentage change) in parental smoking (S1 Table) than in infant’s SHS exposure (Table 2) in each SES level. This suggests that the reduction of infants’ SHS exposure was related to the reduction of parental indoor smoking among smoking parents in addition to the reduction of parental smoking overall.

**Fig 1:**
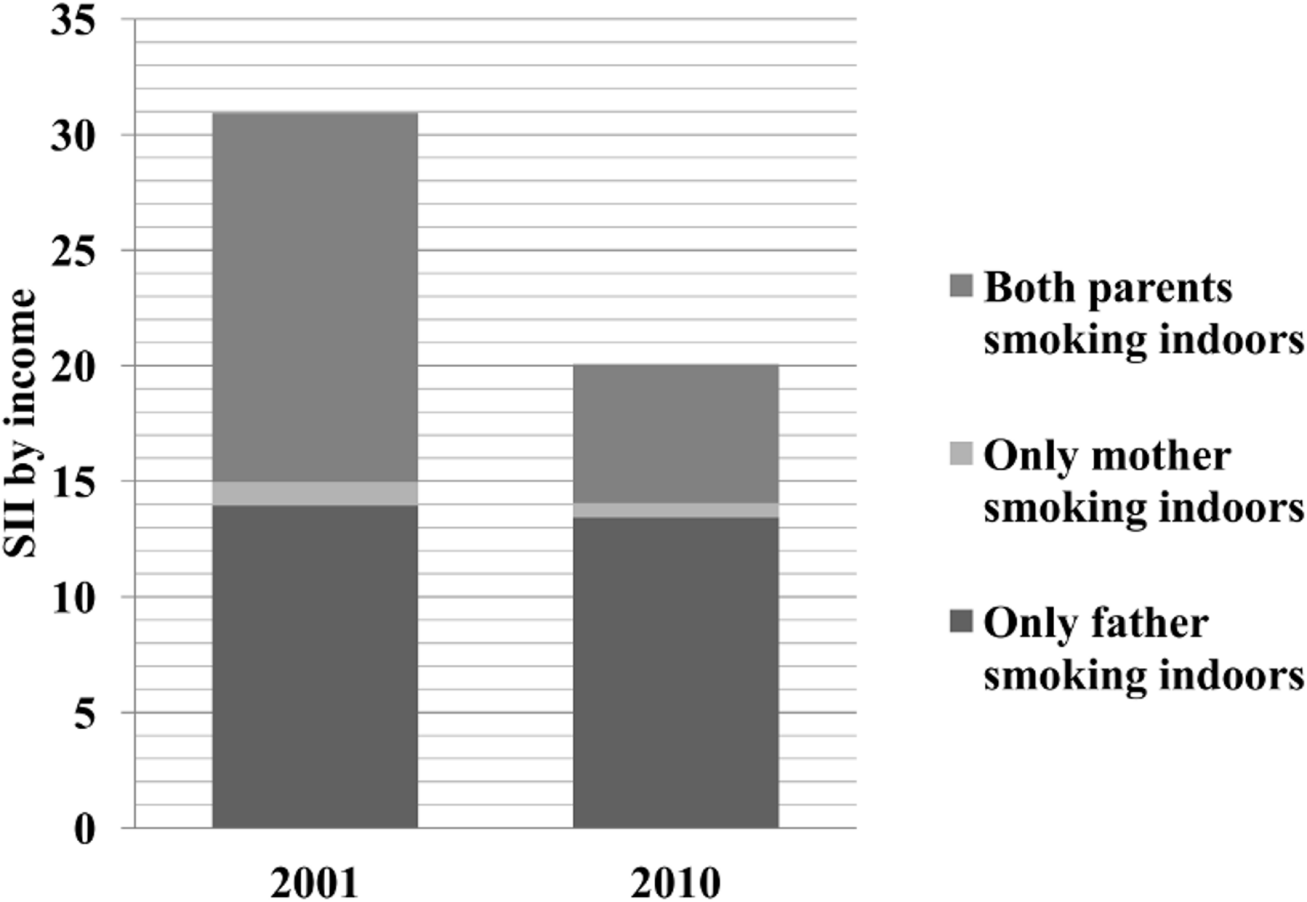
Contributions of parental indoor smoking behaviour to absolute income inequality in SHS exposure in infants. The total bar represents the total absolute income inequality (SII) in each survey year, and each component represents the SII of each parental indoor smoking behaviour.

Regarding SHS exposure from the three parental indoor smoking behaviours, ‘only father smoking indoors’ was a major source of SHS exposure in infants (69.8% in 2001 and 78.7% in 2010) (Table 3). Table 4 shows the prevalence of SHS exposure in infants by parental smoking behaviours according to the income level. Although the overall prevalence of SHS exposure by ‘only father smoking indoors’ decreased by 57.0%, the absolute inequality did not decrease (SII changed from 13.95 in 2001 to 13.45 in 2010) because of the much smaller decrease in the lowest income group (-43.5 percentage change). Fig 1 shows the contributions of absolute income inequality (SII) in SHS exposure in infants to the total income SII by parental indoor smoking behaviour. The proportion represented by ‘only father smoking indoors’ increased (from 45.1% [13.95/30.94] in 2001 to 67.0% [13.45/20.08] in 2010) and became a major contributor in 2010, while the proportion represented by ‘both parents smoking indoors’ decreased over time (from 51.7% [15.99/30.94] in 2001 to 30.0% [6.02/20.08] in 2010).

**Table 3:** Proportion of each parent’s indoor smoking behaviour to the total SHS exposure in infants by survey year.

	2001 (n = 15,403)		2010^a^ (n = 4,619)	
	Number	Proportion	Number	Proportion
Both parents smoking indoors	4,217	27.4	855	18.5
Only father smoking indoors	10,752	69.8	3,635	78.7
Only mother smoking indoors	434	2.8	129	2.8

^a^ The number and proportion in 2010 was weighted for the average parental age in 5-year age groups using a direct method and the age distribution in 2001 as the base.

**Table 4:** Prevalence and magnitude of inequalities in SHS exposure in infants according to the income level by parental smoking behaviour by survey year.

	2001	2010^a^		
**Equivalent household income**	**Prevalence of SHS exposure in infants by both parents smoking indoors (%)**		**Rate difference (%point) (2010–2001)**	**% change ([2010–2001]/2001)**
**Overall**	9.9	2.6	-7.3	-73.4
Quartile 1 (highest) (ref)	4.5	0.6	-3.9	-86.9
Quartile 2	7.6	1.5	-6.1	-79.7
Quartile 3	11.0	2.9	-8.0	-73.3
Quartile 4 (lowest)	16.8	5.2	-11.5	-68.9
**SII (95% CI)**	15.99 (14.92 to 17.05)	6.02 (5.37 to 6.66)		
**RII (95% CI)**	1.61 (1.51 to 1.71)	2.37 (2.17 to 2.57)		
	**Prevalence of SHS exposure in infants by only father smoking indoors (%)**			
**Overall**	25.6	11.0	-14.6	-57.0
Quartile 1 (highest) (ref)	19.5	6.3	-13.3	-68.0
Quartile 2	24.4	8.9	-15.6	-63.7
Quartile 3	29.1	11.6	-17.5	-60.2
Quartile 4 (lowest)	29.6	16.7	-12.9	-43.5
**SII (95% CI)**	13.95 (12.43 to 15.47)	13.45 (12.19 to 14.70)		
**RII (95% CI)**	0.54 (0.49 to 0.60)	1.25 (1.14 to 1.36)		
	**Prevalence of SHS exposure in infants by only mother smoking indoors (%)**			
**Overall**	1.0	0.4	-0.6	-62.9
Quartile 1 (highest) (ref)	0.6	0.2	-0.5	-73.7
Quartile 2	1.0	0.3	-0.7	-69.7
Quartile 3	1.0	0.4	-0.6	-62.3
Quartile 4 (lowest)	1.5	0.7	-0.8	-55.3
**SII (95% CI)**	1.00 (0.64 to 1.36)	0.61 (0.35 to 0.86)		
**RII (95% CI)**	0.98 (0.64 to 1.32)	1.64 (1.02 to 2.26)		

CI, confidence interval; SII, slope index of inequality; RII, relative index of inequality

^a^ The prevalence in 2010 was weighted for the average parental age in 5-year age groups using a direct method and the age distribution in 2001 as the base.

**Fig 2:**
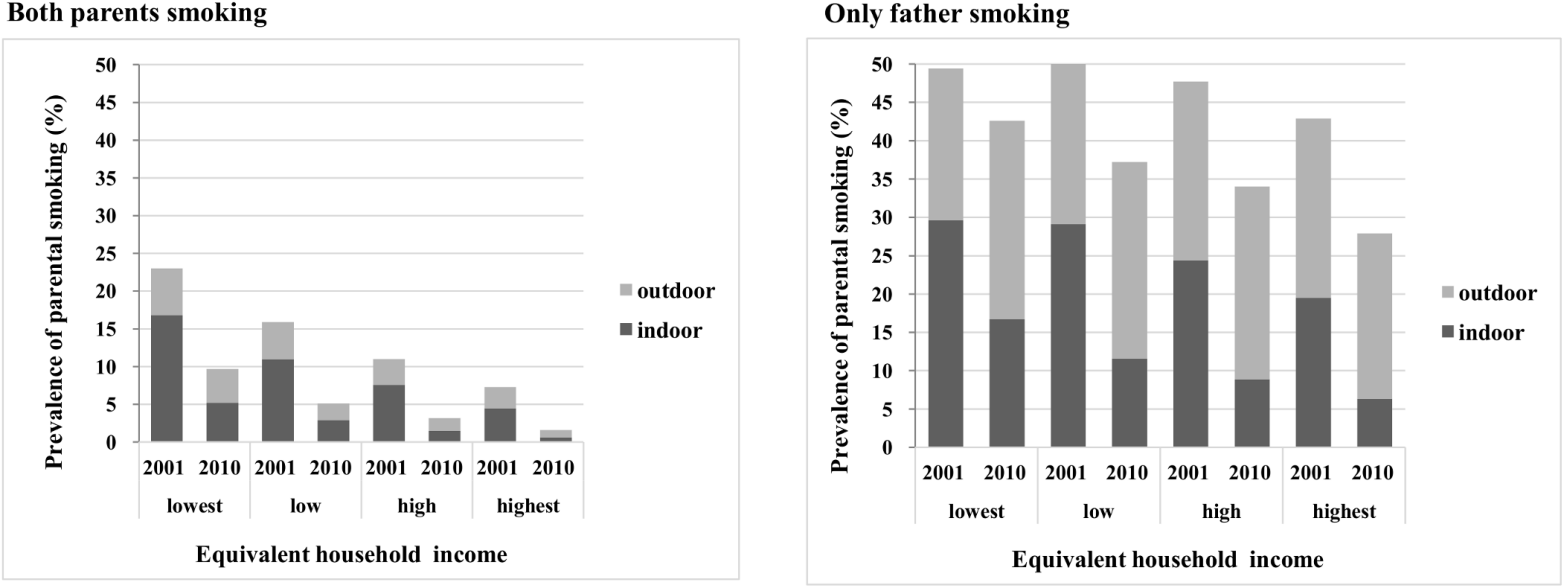
Prevalence of parental smoking and indoor smoking according to the income level by both parents smoking and only father smoking. The prevalence is presented in Table 4. The total bar represents the parental smoking in each survey year, and each coloured bar, dark gray and light gray, represents the parental indoor smoking (SHS exposure in infants) and outdoor smoking, respectively.

By comparing the relative reduction between smoking and indoor smoking by parental smoking behaviours in the sub-analysis (S2 Table, Table 4, Fig 2), we found a much larger difference for ‘only father smoking’ (percentage change, -25.0% for only father smoking vs. -57.0% for only father smoking indoors) than for ‘both parents smoking’ (percentage change, -64.9% for both parents smoking vs. -73.4% for both parents smoking indoors). This suggests that the prevalence of ‘only father smoking indoors’ decreased because not only did the only father smokers decrease, but the indoor smoking among only father smokers also decreased. In contrast, the prevalence of both parents smoking indoors decreased mainly because both parental smokers decreased. Furthermore, the reduction in ‘both parental smoking’ originated mainly from the reduction of the mother smoking, as the relative decrease was as large as both parents smoking (percentage change, -64.9% for both parents smoking and -63.3% for mother smoking) (S2 Table, S3 Table).

## Discussion

In this repeated nationwide population-based survey, we found marked social inequalities in infants’ exposure to SHS in both survey years, with the most exposure occurring for infants in the lowest SES group. From 2001 to 2010, relative inequalities in SHS exposure increased but absolute inequalities in SHS exposure decreased in infants. Furthermore, only father smoking indoors caused 78.7% of infants to be exposed to SHS and it was a major contributor to absolute income inequality in SHS exposure in infants in 2010.

The changes in inequalities in infants’ SHS exposure over time we found in this study are consistent with reports from the USA and England: the SHS exposure inequality there also decreased in absolute but not in relative terms[10,34]. In the USA 7.6% of children are exposed at home (in 2007)[8], in England 12.7% (in 2012)[35], while it is still 14.4% in Japan (in 2010).

The unique finding of this study is that ‘only father smoking indoors’ increased the contribution to the total absolute inequality in infants’ SHS exposure based on income, and it represented the highest contributor in 2010 (Fig 1). The absolute income inequality did not decrease from 2001 to 2010 because the relative reductions for both ‘only father smoking’ and ‘only father smoking indoors’ were the smallest in the lowest income group (S2 Table, Table 4, Fig 2). This might be explained by the low self-efficacy of non-smoking mothers living with a smoking husband in the lowest income group. Low-SES women are less likely to have self-efficacy to avoid SHS exposure than high SES women[36]. Although a mother’s self-efficacy in asking others to smoke outdoors is strongly associated with actual preventive behaviour for their children, non-smoking mothers have a lower self-efficacy than smoking mothers [37].

In contrast, SHS exposure from both parents smoking indoors considerably decrease the absolute income inequality, mainly due to the large reduction in the mother’s smoking. The prevalence of smoking in mothers decreased substantially across all SES levels from 2001 to 2010, compared with that in fathers (percentage change, -34.3% for fathers and -63.3% for mothers) (S3 Table), whereas the prevalence of smoking among men in the general population decreased more than among women during the same periods[20,21]. This is because the prevalence of smoking in women substantially decrease when they become pregnant, although the postpartum relapse rates remain high (approximately 43% at 18 months after childbirth in Japan)[38].

Compared with income, educational inequalities in infants’ SHS exposure appeared to be greater in a similar manner using quartile distribution in both years. In the case of inequalities in parental indoor smoking, nicotine dependence might be a key factor that makes education a stronger predictor of SHS exposure than income. School performance is an indicator of early smoking initiation, which leads to nicotine dependence in later life[39,40]. Thus, compared with income, education level might predict parental nicotine dependence more accurately, and this dependence is one of the main barriers for smoking parents to stop smoking indoors[41,42].

This study showed increases in relative inequalities in infants’ SHS exposure over time, which suggests a need to encourage parents to quit smoking and protect infants from SHS exposure, especially among fathers living with mothers who do not smoke in low-income groups. To reduce father’s (indoor) smoking, targeting non-smoking mothers would be effective for intervention. Compared with fathers, mothers tend to be motivated to protect children from SHS exposure and have many contacts with health professionals[3]. In Japan, local municipalities have implemented “the home visiting program for all households with infants” across the country since 2009. A midwife, nurse, or trained community resident visits homes with infants <4 months old to provide advice regarding child-rearing as well as counselling, and they could provide follow-up services if necessary[43,44]. If tobacco-related issues can be incorporated in this program, it could provide sustainable and individually tailored support to increase self-efficacy in reducing infants’ SHS exposure for low-SES mothers who do not smoke but live with smoking fathers. The program can also encourage smoking fathers and mothers to receive smoking cessation treatments, including nicotine replacement therapy, which have been covered by health insurance in Japan since 2006.

Our study can contribute to strengthen the evidence regarding the inequalities in SHS exposure in infants, particularly regarding the importance of only father smoking in Japan. Nevertheless, the study has certain limitations. First, the exposure to SHS might have been underestimated as parental indoor smoking, a proxy measurement of SHS exposure in infants, did not include exposure from household members other than parents or while outside the home. However, home is known as a primary source of SHS exposure among children[1], and more than 80% of infants in this study did not live with adults other than their parents. Second, the intensity of smoking in the household, such as the number of smokers or number of cigarettes smoked, was not considered[45]. For instance, exposure from mother’s indoor smoking might be more intense than fathers as mothers spend more time at home with their children. Finally, SHS exposure was based solely on parental self-report without biochemical validation, which might be less reliable for populations under pressure not to smoke[46]. Social movements, such as a proposal to decrease children’s passive smoking by the Japan Paediatric Society in 2002[47], might also have influenced under-reporting. However, potential underreporting is not likely to have significantly influenced the changes in inequality as underreporting is not different across SES groups[48].

In conclusion, although the prevalence of SHS exposure in infants decreased considerably from 2001 to 2010 in Japan, inequalities in SHS exposure in infants remained, with increased relative magnitude and decreased absolute magnitude. Further efforts are necessary to encourage parents to quit smoking and protect infants from SHS exposure, especially in low-SES households that include mothers who do not smoke.

## Supporting Information

S1 Table Prevalence of parental smoking and magnitude of inequalities in parental smoking according to the income and educational level by survey year. ^a^ The prevalence in 2010 was weighted for the average parental age in 5-year age groups using a direct method and the age distribution in 2001 as the base. CI, confidence interval; SII, slope index of inequality; RII, relative index of inequality.
(DOCX)

S2 Table Prevalence of parental smoking and magnitude of inequalities in parental smoking according to the income level by combination of parental smoking behavior by survey year. ^a^ The prevalence in 2010 was weighted for the average parental age in 5-year age groups using a direct method and the age distribution in 2001 as the base. CI, confidence interval; SII, slope index of inequality; RII, relative index of inequality.
(DOCX)

S3 Table Prevalence of parental smoking and magnitude of inequalities in parental smoking according to the income level by fathers and mothers by survey year. ^a^ The prevalence in 2010 was weighted for the average parental age in 5-year age groups using a direct method and the age distribution in 2001 as the base.
(DOCX)
