# Supplementary material for: ‘Only Fathers Smoking’ Contributes the Most to Socioeconomic Inequalities: Changes in Socioeconomic Inequalities in Infants’ Exposure to Second Hand Smoke over Time in Japan
Source: PLoS One. 2015 Oct 2;10(10):e0139512. doi: 10.1371/journal.pone.0139512 (PMC4592009; doi:10.1371/journal.pone.0139512)
Supplement: S1 Table — a The prevalence in 2010 was weighted for the average parental age in 5-year age groups using a direct method and the age distribution in 2001 as the base. CI, confidence interval; SII, slope index of inequality; RII, relative index of inequality. (DOCX) [file pone.0139512.s001.docx]

**S1 Table. Prevalence of parental smoking and magnitude of inequalities in parental smoking according to the income and educational level by survey year.**

|  | **Prevalence of parental smoking (%)** | | **Rate difference (%point) (2010 - 2001)** | **% change ([2010-2001]/2001)** |
| --- | --- | --- | --- | --- |
|  | **2001** | **2010^a^** |  |  |
| **Equivalent household income** |  |  |  |  |
| Quartile 1 (highest) (ref) | 51.1 | 29.8 | -21.4 | -41.8 |
| Quartile 2 | 59.8 | 37.6 | -22.2 | -37.1 |
| Quartile 3 | 67.3 | 43.1 | -24.2 | -36.0 |
| Quartile 4 (lowest) | 74.0 | 53.4 | -20.6 | -27.8 |
| **SII (95% CI)** | 30.43 (28.76 to 32.09) | 30.13 (28.19 to 32.07) |  |  |
| **RII (95% CI)** | 0.48 (0.46 to 0.51) | 0.74 (0.69 to 0.79) |  |  |
| **Parental education level** |  |  |  |  |
| Quartile 1 (highest) (ref) | 37.7 | 22.5 | -15.3 | -40.4 |
| Quartile 2 | 51.9 | 33.1 | -18.8 | -36.2 |
| Quartile 3 | 68.1 | 47.4 | -20.7 | -30.4 |
| Quartile 4 (lowest) | 78.2 | 61.7 | -16.5 | -21.1 |
| **SII (95% CI)** | 48.21 (46.62 to 49.79) | 48.46 (46.65 to 50.27) |  |  |
| **RII (95% CI)** | 0.76 (0.74 to 0.79) | 1.19 (1.14 to 1.24) |  |  |

^a^ The prevalence in 2010 was weighted for the average parental age in 5-year age groups using a direct method and the age distribution in 2001 as the base.

CI, confidence interval; SII, slope index of inequality; RII, relative index of inequality
