# Supplementary material for: ‘Only Fathers Smoking’ Contributes the Most to Socioeconomic Inequalities: Changes in Socioeconomic Inequalities in Infants’ Exposure to Second Hand Smoke over Time in Japan
Source: PLoS One. 2015 Oct 2;10(10):e0139512. doi: 10.1371/journal.pone.0139512 (PMC4592009; doi:10.1371/journal.pone.0139512)
Supplement: S2 Table — a The prevalence in 2010 was weighted for the average parental age in 5-year age groups using a direct method and the age distribution in 2001 as the base. CI, confidence interval; SII, slope index of inequality; RII, relative index of inequality. (DOCX) [file pone.0139512.s002.docx]

**S2 Table. Prevalence of parental smoking and magnitude of inequalities in parental smoking according to the income level by combination of parental smoking behavior by survey year.**

|  | **Prevalence of both parents smoking (%)** | | **Rate difference (%point) (2010 - 2001)** | **% change ([2010-2001]/2001)** |
| --- | --- | --- | --- | --- |
|  | **2001** | **2010^a^** |  |  |
| **Overall** | 14.2 | 5.0 | -9.2 | -64.9 |
| Quartile 1 (highest) (ref) | 7.3 | 1.6 | -5.7 | -78.4 |
| Quartile 2 | 11.0 | 3.2 | -7.9 | -71.2 |
| Quartile 3 | 15.9 | 5.1 | -10.8 | -68.0 |
| Quartile 4 (lowest) | 23.0 | 9.7 | -13.4 | -58.0 |
| **SII (95% CI)** | 20.74 (19.51 to 21.97) | 10.30 (9.42 to 11.17) |  |  |
| **RII (95% CI)** | 1.46 (1.38 to 1.54) | 2.13 (1.98 to 2.29) |  |  |
|  | **Prevalence of only father smoking (%)** | |  |  |
| **Overall** | 47.6 | 35.6 | -11.9 | -25.0 |
| Quartile 1 (highest) (ref) | 42.9 | 27.9 | -15.0 | -34.9 |
| Quartile 2 | 47.7 | 34.0 | -13.7 | -28.8 |
| Quartile 3 | 50.4 | 37.2 | -13.2 | -26.1 |
| Quartile 4 (lowest) | 49.4 | 42.6 | -6.8 | -13.8 |
| **SII (95% CI)** | 8.89 (7.13 to 10.65) | 18.73 (16.83 to 20.63) |  |  |
| **RII (95% CI)** | 0.19 (0.15 to 0.22) | 0.53 (0.48 to 0.58) |  |  |
|  | **Prevalence of only mother smoking (%)** | |  |  |
| **Overall** | 1.2 | 0.7 | -0.5 | -41.7 |
| Quartile 1 (highest) (ref) | 0.9 | 0.3 | -0.7 | -71.5 |
| Quartile 2 | 1.1 | 0.5 | -0.6 | -55.9 |
| Quartile 3 | 1.1 | 0.8 | -0.3 | -24.7 |
| Quartile 4 (lowest) | 1.6 | 1.1 | -0.4 | -27.9 |
| **SII (95% CI)** | 0.84 (0.45 to 1.22) | 1.07 (0.73 to 1.41) |  |  |
| **RII (95% CI)** | 0.71 (0.39 to 1.04) | 1.59 (1.15 to 2.04) |  |  |

^a^ The prevalence in 2010 was weighted for the average parental age in 5-year age groups using a direct method and the age distribution in 2001 as the base.

CI, confidence interval; SII, slope index of inequality; RII, relative index of inequality
