# Supplementary material for: ‘Only Fathers Smoking’ Contributes the Most to Socioeconomic Inequalities: Changes in Socioeconomic Inequalities in Infants’ Exposure to Second Hand Smoke over Time in Japan
Source: PLoS One. 2015 Oct 2;10(10):e0139512. doi: 10.1371/journal.pone.0139512 (PMC4592009; doi:10.1371/journal.pone.0139512)
Supplement: S3 Table — a The prevalence in 2010 was weighted for the average parental age in 5-year age groups using a direct method and the age distribution in 2001 as the base. (DOCX) [file pone.0139512.s003.docx]

**S3 Table. Prevalence of parental smoking and magnitude of inequalities in parental smoking according to the income level by fathers and mothers by survey year.**

|  | **Prevalence of father smoking (%)** | | **Rate difference (%point) (2010 - 2001)** | **% change ([2010-2001]/2001)** |
| --- | --- | --- | --- | --- |
|  | **2001** | **2010^a^** |  |  |
| **Overall** | 61.8 | 40.6 | -21.2 | -34.3 |
| Quartile 1 (highest) (ref) | 50.2 | 29.5 | -20.7 | -41.2 |
| Quartile 2 | 58.7 | 37.1 | -21.6 | -36.7 |
| Quartile 3 | 66.3 | 42.3 | -24.0 | -36.2 |
| Quartile 4 (lowest) | 72.4 | 52.3 | -20.2 | -27.8 |
|  | **Prevalence of mother smoking (%)** | | **Rate difference (%point) (2010 - 2001)** | **% change ([2010-2001]/2001)** |
|  | **2001** | **2010^a^** |  |  |
| **Overall** | 15.4 | 5.7 | -9.7 | -63.3 |
| Quartile 1 (highest) (ref) | 8.2 | 1.8 | -6.4 | -77.6 |
| Quartile 2 | 12.2 | 3.7 | -8.5 | -69.8 |
| Quartile 3 | 17.0 | 5.9 | -11.1 | -65.4 |
| Quartile 4 (lowest) | 24.6 | 10.8 | -13.8 | -56.1 |

^a^ The prevalence in 2010 was weighted for the average parental age in 5-year age groups using a direct method and the age distribution in 2001 as the base.
